# Right ventricular failure: Current strategies and future development

**DOI:** 10.3389/fcvm.2023.998382

**Published:** 2023-04-28

**Authors:** María Monteagudo-Vela, Alexander Tindale, Emilio Monguió-Santín, Guillermo Reyes-Copa, Vasileios Panoulas

**Affiliations:** ^1^ Cardiothoracic Surgery Department, Hospital Universitario de la Princesa, Madrid, Spain; ^2^ Department of Cardiothoracic Transplantation and Mechanical Circulatory Support, Royal Brompton and Harefield Hospitals, Guy’s and St Thomas’ NHS Foundation Trust, London, United Kingdom; ^3^ Department of Cardiology, Royal Brompton and Harefield Hospitals, Guy’s and St Thomas’ NHS Foundation Trust, London, United Kingdom; ^4^ Cardiovascular Sciences, National Heart and Lung Institute, Imperial College London, London, United Kingdom

**Keywords:** right ventricular failure, right catheterization, impella, haemodynamic, future management

## Abstract

Right heart failure can be defined as a clinical syndrome consisting of signs and symptoms of heart failure resulting from right ventricular dysfunction. Function is normally altered due to three mechanisms: (1) pressure overload (2) volume overload, or (3) a decrease in contractility due to ischaemia, cardiomyopathy or arrythmias. Diagnosis is based upon a combination of clinical assessment plus echocardiographic, laboratory and haemodynamic parameters, and clinical risk assessment. Treatment includes medical management, mechanical assist devices and transplantation if recovery is not observed. Distinct attention to special circumstances such as left ventricular assist device implantation should be sought. The future is moving towards new therapies, both pharmacological and device centered. Immediate diagnosis and management of RV failure, including mechanical circulatory support where needed, alongside a protocolized approach to weaning is important in successfully managing right ventricular failure.

## Introduction

Right heart failure (RHF) can be defined as a clinical syndrome consisting of signs and symptoms of heart failure resulting from right ventricular dysfunction (RVD) (1).

At its core is a progressive syndrome of systemic congestion when the right ventricle (RV) cannot meet blood flow demands without excessive use of the Frank–Starling mechanism (2). The function is usually by either pressure or volume overload, although a direct decrease in contractility can play a role. Whatever the aetiology, RVD is associated with increased morbidity and mortality.

The prevalence of RVF is often underestimated as it has been historically underdiagnosed. However, the most common causative etiologies are well documented. It is observed in 3%–9% of acute heart failure admissions, and the in-hospital mortality of patients with acute RV failure ranges from 5% to 17% (3). In the special case of RVF after LVAD implantation, the prevalence ranges from 9% to 40% (4).

Patients are especially vulnerable to RVD if they are more likely to experience sudden changes in preload (e.g., renal replacement therapy or sepsis) or afterload (e.g., long-term ventilation or coagulation disorders) (5).

Diagnosis of RV failure is more subtle than overt left ventricular dysfunction and is thus often delayed, which worsens the prognosis. The combination of clinical risk assessment and the use of existing diagnostic methods such as echocardiography, MRI (Magnetic Resonance Imaging), haemodynamic and biochemical markers are the key to accurate, early diagnosis and successful management.

Treating RV failure should be targeted at correcting the underlying mechanism and therefore revolves around maintaining adequate perfusion pressure, optimizing the preload and reducing the afterload with the aim of improving the cardiac output. Methods for fulfilling these goals include respiratory and mechanical circulatory support in addition to medical therapy.

### History

Up until the second half of the 20th century, the study of the heart was disproportionally focused on the left ventricle (LV) with the RV being largely neglected. In the early 1980s, a growing interest in the study of the pathophysiology of the RV emerged, leading to the development of new imaging techniques, different surgical approaches, and an increase in the research of hemodynamic parameters and study of the ventricular interdependence (5). The appearance of special haemodynamic situations, such as the management in the setting of a Heart Transplantation (HTx) or Left Ventricular Assist Device (LVAD) implantation, increased the incentive to understand the pathophysiology of RV failure.

Recent advances in echocardiography and MRI have expanded the knowledge in the field of the anatomy and physiology of the RV and has shown how the mechanisms of left and right ventricular failure are very different. However, despite these advances there remain relatively few direct treatment strategies and few evidence-based interventions available than for its left-sided counterpart.

## Pathophysiology

The normal function of the right ventricle is the result of interplay between venous return (preload), PA pressures (afterload), right ventricular myocardial contractility, pericardial compliance and interventricular dependence (5). As such, changes to any of these physiological parameters can result in RV failure.

### Acute right ventricular failure

Acute RV failure can therefore be divided based upon the underlying physiologic mechanism, and these are summarized in Table 1.

**Table 1:** Causes of right ventricular failure.

Acute RVF
Volume overload
Acute left-sided heart failure
LVAD implantation
Pressure overload
Acute pulmonary embolism
Hematological disorders (e.g., acute chest syndrome in sickle cell disease)
Decreased contractility
Acute myocardial ischemia
Fulminant myocarditis
Pericardial disease (tamponade)
Sepsis (can cause increased venous return and volume overload)
Post-cardiotomy shock
Reduced pericardial compliance
Chronic RHF
Exacerbation of chronic lung disease and/or hypoxia
Chronic pulmonary hypertension (groups 1–5)
Pericardial disease (constrictive pericarditis)
Arrhythmias (supraventricular or ventricular tachycardia)
Congenital heart disease (e.g., atrial or ventricular septal defect, Ebstein's anomaly)
Valvulopathies (e.g., tricuspid valve regurgitation, pulmonary valve stenosis)
Cardiomyopathies (e.g., arrhythmogenic right ventricular dysplasia, familial, idiopathic)
Myocarditis or other inflammatory diseases

Increased pulmonary pressures leads to **pressure overload**, caused by either primary problems of the pulmonary vasculature (e.g., pulmonary embolism—PE) or as a result of acute left sided failure. The structure of the RV makes it poorly adapted to abrupt increases in upstream pressures and hence in the acute phase, the right ventricle cannot easily adapt in a homeometric fashion.

The pulmonary vasculature is high-compliance (i.e., it can accept large changes in volume) but low pressure. As LaPlace's law would suggest, the RV is well suited to these conditions by being thinner-walled and more compliant than the LV. This leaves it exquisitely sensitive to increased afterload. A small increase in afterload can cause a large reduction in stroke volume without an increase in pressure generation (5).

Therefore, the RV does not cope well with acute increases in upstream pressures—and RV ejection fraction (RVEF) is inversely proportional to pulmonary arterial systolic pressure (PASP), and hence increased afterload, in a manner which is not replicated for the left ventricle (6). Adjustment of afterload is key in the management of RV dysfunction.

Increased venous return can cause **volume overload**, progressive dilatation and dysfunction. However, in contrast to the response to pressure overload, the RV can adapt efficiently in a heterometric manner and hence copes well with volume overload. Examples of acute RV failure from volume overload include LVAD implantation or aggressive fluid resuscitation, especially in high output states such as sepsis.

Sudden **reductions in contractility** are often caused by myocardial damage, as seen in RV infarction or myocarditis, or a more global rhythm disturbance. This leads to reduced stroke volume and progressive RV dilatation, which can precipitate a negative spiral into tricuspid regurgitation, further dilatation and LV impingement due to interventricular dependence (5).

This concept of **interventricular dependence** is especially important when assessing the right ventricle. This can take the form of either systolic or diastolic interaction. In the former, the interaction between the left and right sides is due to the contraction of one ventricle assisting contraction of the other. Due to relative muscle mass this effect is more pronounced for the RV: up to 40% of RV systolic pressure generation is due to LV contraction (7). In contrast, diastolic interdependence occurs when the ventricles compete for filling space. This can occur with non-dilated cavities when the pericardium is non-compliant, such as occurs acutely in cardiac tamponade (8) but can also occur when RV pressure overload leads to diastolic filling disruption in the LV, as occurs in large pulmonary emboli precipitating low cardiac output states.

These pathophysiologic mechanisms do not exist in isolation; rather they frequently co-exist. For example, a large posterior myocardial infarction can cause acute mitral regurgitation (MR), increasing pulmonary pressures, as well as reducing myocardial contractility, and worsen RV function due to interaction between the left and right ventricles.

### Chronic right ventricular failure

The causes of chronic RV failure can be similarly subdivided by the causative mechanism.

Any conditions causing chronic raised pulmonary pressures will increase the afterload. This includes primary causes of pulmonary hypertension as well as chronic left-sided heart failure. Patients with right heart failure secondary to increased pulmonary pressures have a poor prognosis (8).

Volume overload as a cause of chronic right heart failure is often due to shunts, such as congenital heart disease, or right-sided valvular regurgitation. Chronic right heart failure due to either pressure or volume overload follows a similar course. There is initially a compensatory phase that involves hypertrophy and fibrosis. As the disease progresses there are various metabolic changes including mitochondrial dysfunction and fibrosis that leads to progressive RV dysfunction and dilatation (9).

As the RV dilates and becomes dysfunctional, it impinges on the LV and causes reduced left-sided cardiac output. However, in chronic pericardial diseases the reduction in left sided cardiac output does not require RV dilatation; rather there is a competition for space within a fixed volume pericardium.

Finally, several cardiomyopathies can lead directly to reduced RV function by directly affecting contractility, including arrhythmogenic right ventricular dysplasia (ARVD), dilated cardiomyopathies and the long-term sequelae of myocarditis.

## Diagnosis

Early diagnosis is key in successful management of RV dysfunction, but it is often delayed or missed, possibly because the clinical signs can be more subtle than LV dysfunction. It is necessary to use a combination of clinical, echocardiographic, laboratory and hemodynamic parameters to accurately recognize the presence and underlying mechanism of RV failure.

**Figure 1:**
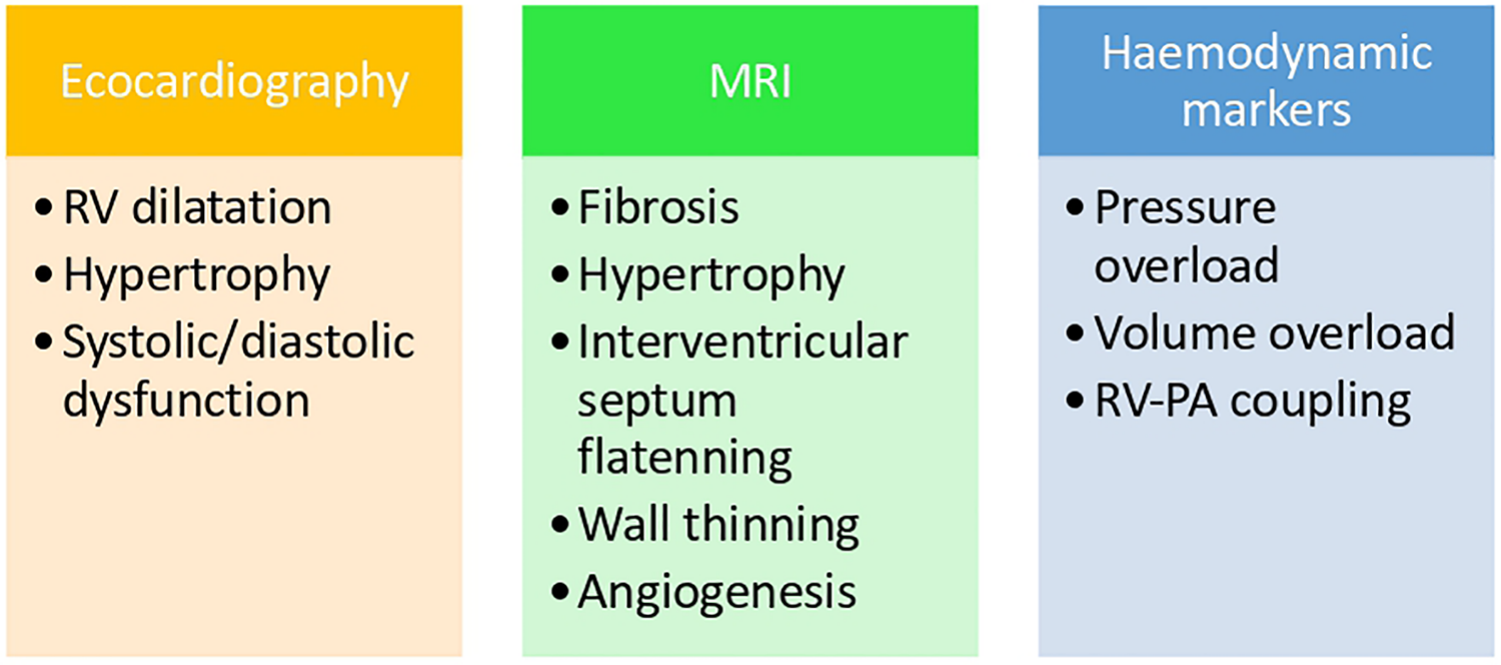
Correlation between pathophysiological mechanism and diagnostic tools.

Figure 1 shows the relationship between the diagnosis tools and the pathophysiological mechanisms explained earlier.

### Echocardiography

Echocardiography should be the first-line test in the assessment of acute RV failure because it is fast, non-invasive, and can identify life-threatening acute pathologies such as tamponade and PE.

There are several parameters that can help with the assessment of the right ventricle.

Tricuspid annular plane systolic excursion (TAPSE) is the most used and well-established parameter to evaluate RV systolic function, however, it only assesses longitudinal contraction, hence giving us only partial information about the RV function. For example, TAPSE is not suitable after tricuspid valve annuloplasty as it leads to an underestimation of RV systolic function (10). Similarly, using tissue doppler parameters such as RV S' also offer only longitudinal assessment.

**Figure 2:**
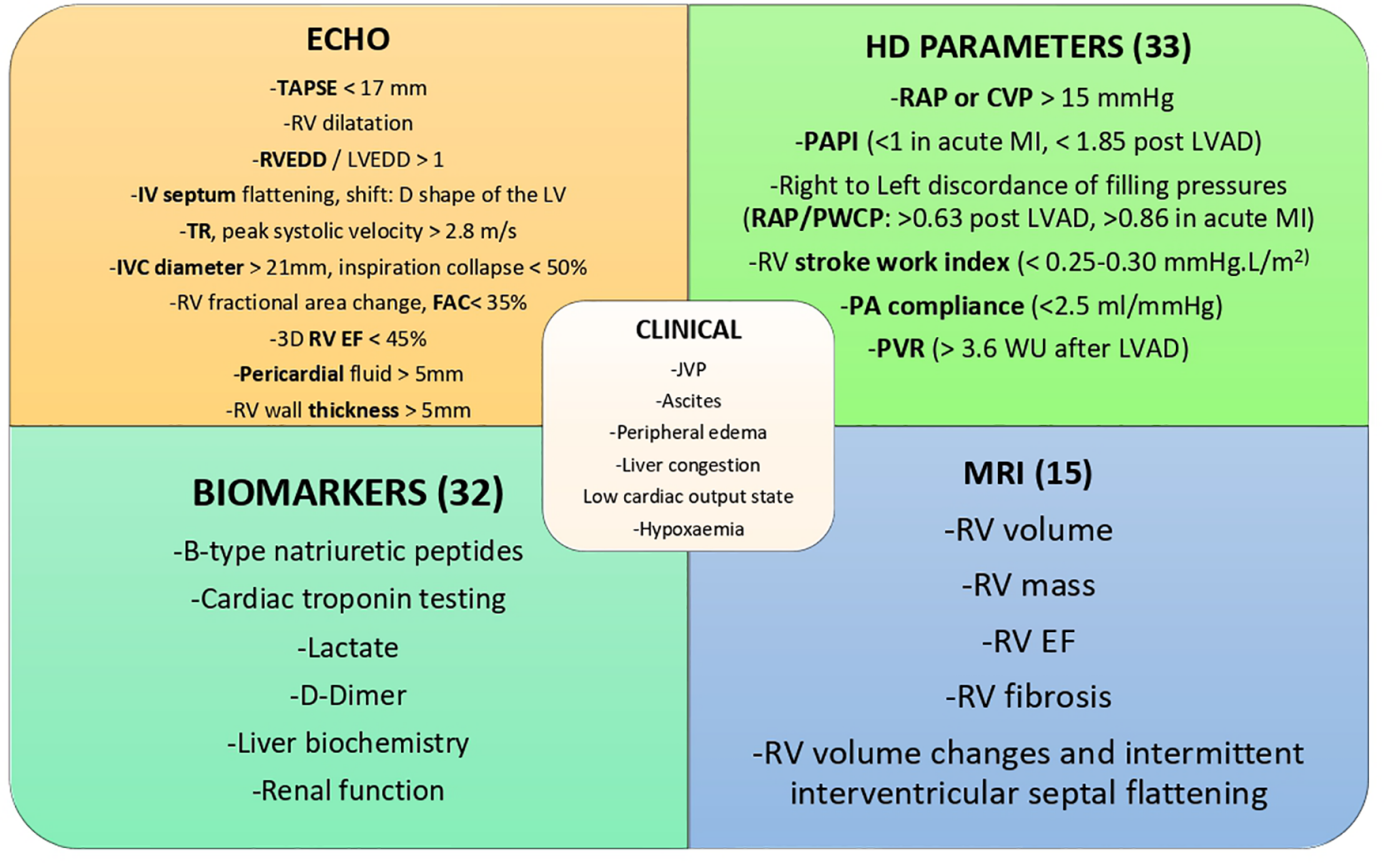
ECHO; echocardiography parameters: TAPSE, tricuspid annular plane systolic excursion; RVEDD, right ventricular end-diastolic diameter; LVEDD, left ventricular end-diastolic diameter; IV, interventricular; LV, left ventricle; TR, tricuspid regurgitation; IVC, inferior vena cava; RV, right ventricle/ventricular; FAC, fractional area change; EF, ejection fraction; HD, haemodynamic parameters; RAP, right atrial pressure; CVP, central venous pressure; PAPI, pulmonary artery pulsatility index; MI, myocardial infarction; LVAD, left ventricular assist device; PWCP, pulmonary capillary wedge pressure, PA, pulmonary artery; PVR, pulmonary vascular resistance; JVP, jugular venous pressure; MRI, magnetic resonance imaging.

New echo techniques are shifting from qualitative to quantitative evaluation (11). The most frequently used parameters of RV function, size and area are represented in Figure 2. Both RV strain and 3D RV chamber quantification have been shown to have strong prognostic capabilities (12). There has also been a recent focus on using markers of venous congestion as a surrogate marker of RV function, which is especially useful in guiding fluid administration in the intensive care setting (13).

As discussed previously, pressure readings are crucial in diagnosis of acute RV failure. These include estimating PASP by utilizing a combination of peak TR velocity, right atrium (RA) size and inferior vena cava (IVC) dimensions, as well as pulmonary valve (PV) acceleration time.

Visually, ventricular interdependence and septal bounce are important clinical signs, especially in the context of pericardial disease processes.

Once right ventricle dysfunction has been diagnosed, echocardiography is also the most crucial tool in hunting for the underlying aetiology, specifically common left heart failure and valvular abnormalities. But it can also provide valuable information regarding evolution of the disease and the impact of treatment.

### MRI

As a 3-dimensional, tomographic imaging modality with good temporal and spatial resolution, MRI does offer advantages over echocardiography and hence has become the gold standard for assessing the morphological characteristics of RV function. It is more accurate than echocardiography in measuring these parameters, but this is only a marginal improvement because modern echo is accurate and is more sensitive at assessing pulmonary pressures. What sets cardiac MRI apart is the ability to accurately characterize tissue properties (14). Therefore, if the cause of right heart failure is due to reduced myocardial contractility, cardiac MRI can differentiate clearly and accurately between infarction, myopathies such as ARVD (Arrhythmogenic Right Ventricle Dysplasia), or myocarditis, and hence direct the treatment accordingly.

Apart from the myocardium, cardiac MRI also gives very detailed imaging to differentiate between conditions causing changes to the pre-and after load, such as congenital heart defects as a cause of pulmonary hypertension (PH), or chronically thickened pericardium. Velocity-encoded imaging adds to the structural information to calculate flow through valves or shunts (15), which can be used prognostically when assessing PH (16).

Cardiac MRI does require expensive scanning equipment, dedicated radiographers, is unsuitable for claustrophobic patients or those who cannot breath-hold adequately, and patients with older pacemakers (17). However, almost all implanted pacemakers in the UK and Europe are now MRI compatible, but defibrillators will require a dedicated cardiac physiologist to deactivate certain therapies (such as anti-tachycardia therapies) during the scan (18).

Therefore, Cardiac MRI offers excellent characterization of tissue and overall cardiac structure. While MRI can assess flow through structures, it is best use as a complimentary adjunct to, rather than a replacement for, echocardiography.

### Biomarkers

There are no specific biomarkers for the diagnosis of RV failure. The levels of frequently used biomarkers, such as B-Natriuretic Peptide (BNP) or cardiac troponins depend on the underlying pathology and clinical context in which RV failure is presented. New biomarkers more specific for the diagnosis of RV failure are currently under research (19).

### Hemodynamic markers

Invasive hemodynamic measurement with a pulmonary artery (PA) catheter is the most accurate method of assessing right-sided filling pressures, contractility, and afterload. It provides continuous, real-time data; however, it should be used for the shortest time possible.

Parameters such as cardiac filling pressures, PA pulsatility index (PAPi), RV stroke work index (RVSWI), Pulmonary Vascular Resistance (PVR) and transpulmonary gradient (TPG) offer prognostic information in patients with RVF (Figure 2). PAPi, defined as PA pulse pressure/mean right atrial pressure (mRAP), has shown prognostic value in a variety of patient cohorts. These include assessment after LVAD insertion, advanced heart failure populations including patients with pulmonary arterial hypertension (PAH) and after acute myocardial infarction (20, 21). Recently it has been shown to be a predictor of mortality and major adverse cardiovascular events in a very heterogeneous hospitalized population (22). In the latter study there did not appear to be a specific cut-off but rather a graded association of PAPi quartiles and mortality, with those at the lowest faring worse.

RV stroke work index (RVSWI) is another prognostic hemodynamic parameter. It is calculated by the following equation: RVSWI = SVI × (mPA − mRAP) × 0.0136, where stroke volume index (SVI) is calculated by the [cardiac index/heart rate] × 1,000 (23, 24). A low RVSWI has been associated with worse outcomes in several studies (25).

As discussed in the pathophysiology section, afterload is extremely important in assessing RV failure. However, pulmonary afterload is not a static process—as the RV contracts, the compliance of the pulmonary arteries exerts an opposing force.

Hence pulmonary afterload encompasses both a “static” element (which is the PVR) and a dynamic component (which includes compliance (PC) and characteristic impedance) (26).

Often, PVR has been used as a proxy for afterload. However, it only accounts for around 75% of pulmonary afterload (26). PVR is defined as the difference between mean PA pressure and pulmonary capillary wedge pressure (PCWP) divided by cardiac output. Some attempts have been made to find proxy measurements using echocardiography, such as the Peak TR velocity: LVOT VTI ratio but are not accurate enough to replace invasive measurements.

PC accounts for around 20% of pulmonary afterload and can be estimated by the ratio of stroke volume to PA pulse pressure measured by right heart catheterization (27). Loss of compliance is associated with factors that occur during worsening of PH such as elastin and collagen reduction in proximal PAs (28, 29) and is associated with parameters of RV failure including hypertrophy and systolic dysfunction (30).

**Figure 3:**
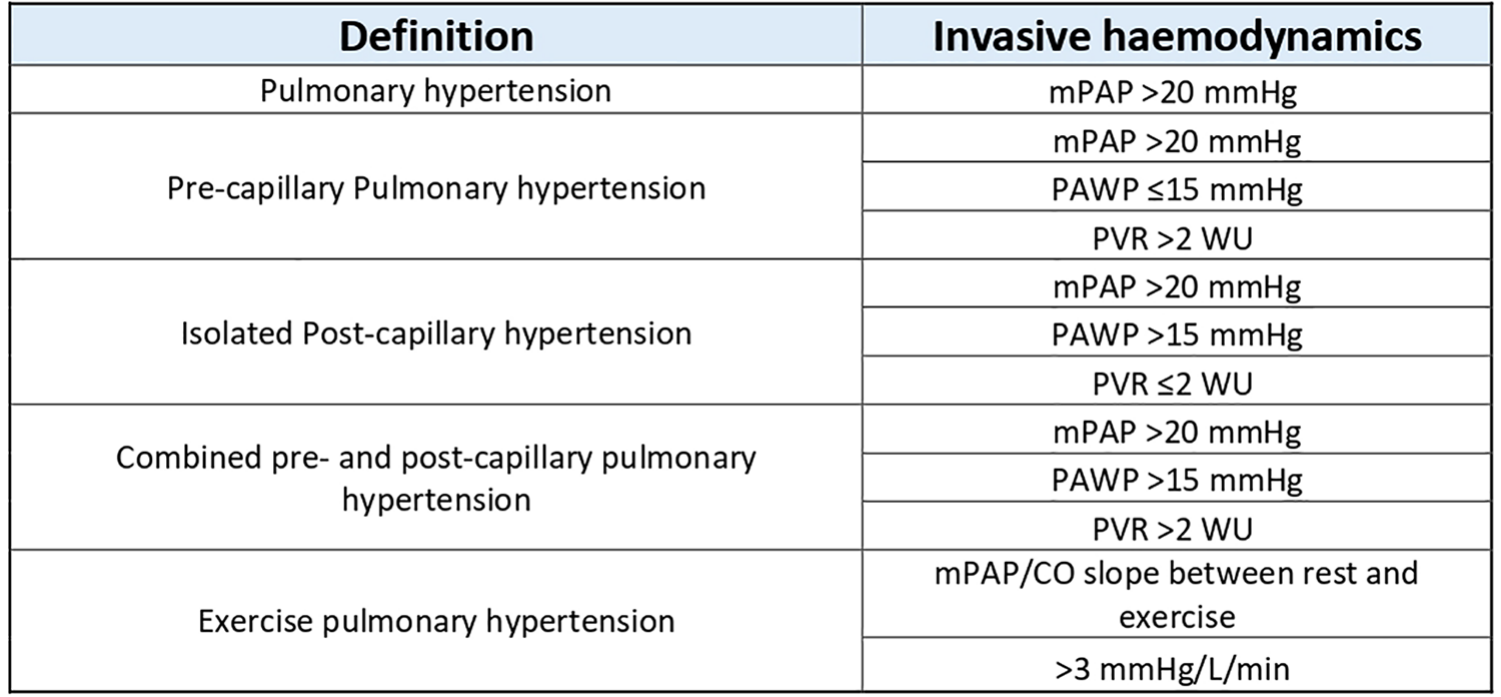
Definitions and categorisation of PH using invasive haemodynamics. Table reproduced from Humbert 2022 (ESC PH guidelines) (31).

TPG allows the determination of the cause of right heart failure if it coexists with pulmonary hypertension, where a high TPG (>12 mmHg) and a low PCWP (<15 mmHg) point to a pre-capillary cause and the reverse situation (TPG <12 mmHg and PCWP >15 mmHg) point to a left-sided aetiology. A high TPG and high PCWP co-exists in combined pre- and post-capillary PH (Figure 3) (31).

**Figure 4:**
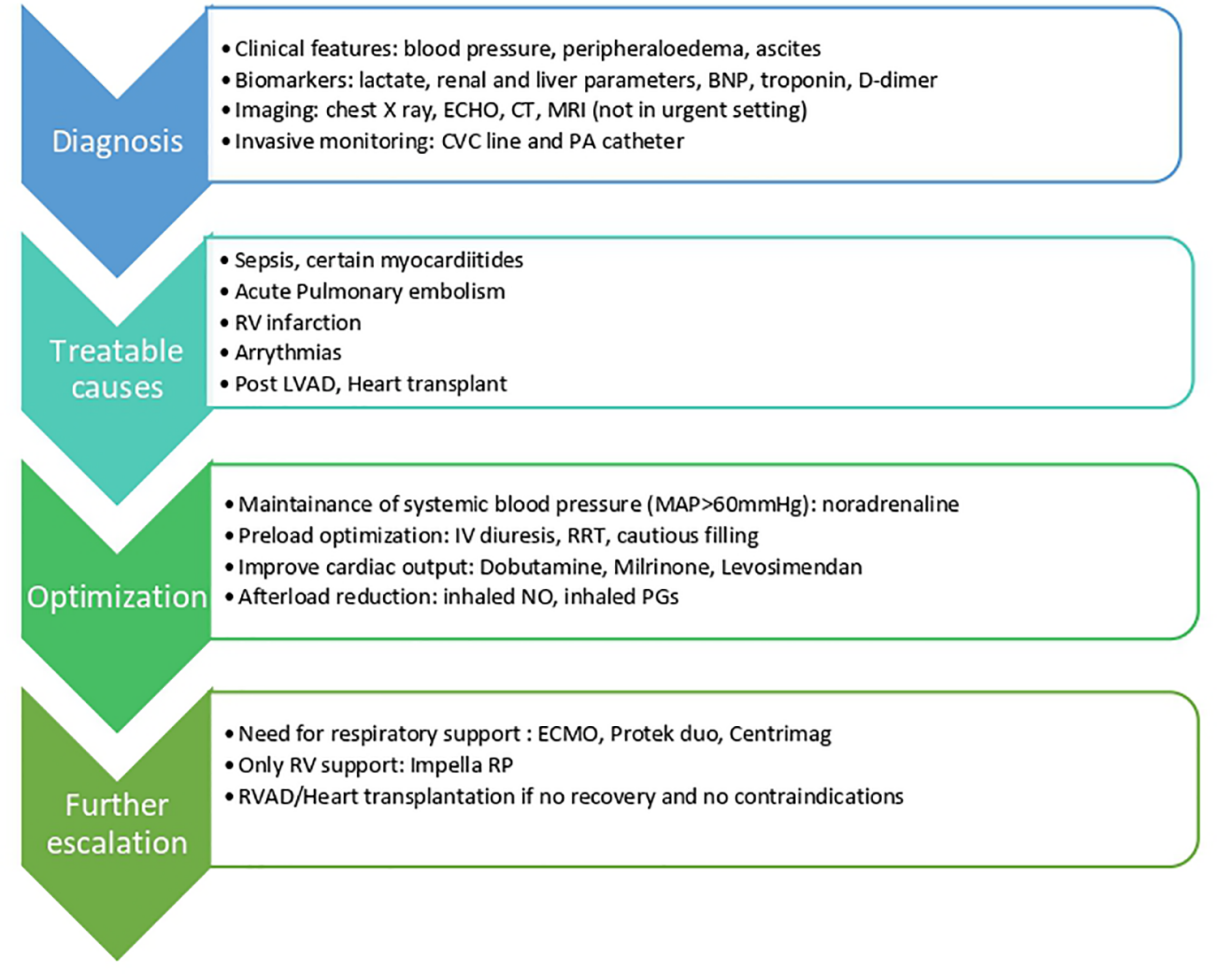
ECHO; echocardiography parameters; CT, computered tomography; MRI, magnetic resonance imaging, CVC, central venous catheter; PA, pulmonary artery; RC, right ventricle; LVAD, left ventricular assist device; IV, intravenous; MAP, mean arterial pressure; RRT, renal replacement therapy; NO, nitric oxide; PGs, prostaglandins; ECMO, extracorporeal membrane oxygenation; RVAD, right ventricular assist device.

Finally, the concept of RV-PA coupling unifies many of the points made above. The morphological adaptation of the RV to increasing pulmonary pressures can only compensate to a certain point (RV-PA coupling) before the RV can no longer compensate. Several studies combining invasive haemodynamics with echocardiographic markers of RV function have shown this combination to be more predictive than RV function alone (32, 33).

## Treatment

The management of RVF depends on the underlying mechanism, for example thrombolysis if the cause is acute PE. However, the overall management requires a similarly thorough diagnostic evaluation and there is considerable overlap between the therapeutic approaches (Figure 4).

1. **Medical management**

The medical management of RVF encompasses a combination of pharmacologic and non-pharmacologic treatments to optimize preload, reduce afterload with the goal of enhancing RV contractility. Invasive monitoring *via* a Swan-Ganz catheter gives critical information to allow timely treatment titration and escalation (5).

**Management of acute RHF**

1. Management of specific conditions

The diagnosis of specific conditions allows accurate and tailored management. The most common specific causes of right heart failure are:

(i) RV infarction—treated with reperfusion and fluid resuscitation
(ii) Pulmonary Embolus: anticoagulation and, if indicated, thrombolysis or clot retrieval
(iii) Critically unwell patients: a homogenous group who require careful volume management with a high output state into a vasodilated system—can easily result in volume overload—see “Preload optimization” section below. If ventilated, high inspiratory pressures increase RV afterload, hence keep inspiratory pressure as low as feasible.

2. Preload optimization

Although increases in afterload are often the primary cause of RV failure, preload optimization is the initial target for therapy because it is fast to initiate treatment, easy to monitor and has clear goal-directed targets and is effective due to its interaction and effect on RV afterload.

Therefore, volume optimization is key, whether the patient requires volume initially (such as RV infarct) or is volume overloaded. At a minimum, care should be directed towards maintaining a central venous pressure of 8–12 mmHg (5).

Historically, the RV was viewed as a passive conduction system and hence was often managed by increasing volume and right atrial pressures to increase pulmonary circulation. However, if the RV begins dilating, especially in the face of increased afterload, wall stress increases rapidly in such a thin-walled structure. This leads to further dilatation, reduction in systolic pressure generation, increased TR and impaired LV filling due to aforementioned interventricular dependence. Myocardial ischemia, due to both reduced CO and increased RV wall stress, and congestive multiorgan failure can ensue (34).

The easiest and most effective method to reduce RV preload is using a high-dose furosemide infusion to reduce right atrial pressure to between 8 and 12 mmHg. The response must be rapidly assessed to allow escalation in therapy or addition of thiazides, which potentiate loop diuretics. Clearly, regular electrolyte monitoring is mandatory due to the possibility of rapid shifts. The addition of carbonic anhydrase inhibitors can improve any alkalosis resulting from loop and thiazide diuretics (35).

Renal replacement therapy (RRT) is effective at removing excess fluid. Although commonly initiated in diuretic resistant patients, there is no clear evidence supporting their use in acute RV failure. Thus, RRT should not be considered first-line treatment but started after failure of diuretic therapy. It should be conducted at a rate that allows an adequate shift of extravascular to intravascular space (5).

In contrast, intravenous fluids are required in cases of associated hypovolemia to reach the above-mentioned target, for example in cases of RV infarction. However, if increased afterload is the primary cause of RV failure, even if the patient is volume deplete, this must be performed carefully and in small increments otherwise decompensation is likely.

3. Afterload reduction

Specific afterload reduction therapies depend on the specific pathophysiology, such as in pulmonary emboli or increased ventilation, as discussed previously. In the absence of a specific reversible cause, pulmonary vasodilators decrease pulmonary vascular bed resistance leading to a decrease of RV afterload.

Intravenous agents such as milrinone (see below) are effective at both causing pulmonary vasodilation and increased contractility, although it causes both pulmonary and systemic vasodilation and hence can enhance hypotension.

Selective pulmonary vasodilators include inhaled or parenteral epoprostenol (36, 37) or inhaled nitric oxide. The principal benefit of inhaled agents is the absence of systemic adverse effects, mainly hypotension. Nitric oxide administration (5–20 ppm) has showed to be beneficial in terms of reduction of pulmonary vascular resistance and increase of RV ejection fraction (38). Oral phosphodiesterase-5 inhibitors have been used in RVF after LVAD implantation in some small series (39).

They should not be administered in patients with postcapillary pulmonary hypertension and RV failure before the left sided problem (LV dysfunction, valve disease) has been addressed. Increasing transpulmonary flow in patients with elevated left atrial pressure may induce pulmonary oedema.

4. RV contractility enhancement

Regardless of the underlying cause of acute RHF, the endpoint is increased wall stress and reduced myocardial contractility due to both direct (such as overstretched myocytes) and indirect mechanisms (including reduced perfusion and reduction in LV function/output).

Inotropic agents, such as dobutamine or milrinone, that increase myocardial contractility and RV cardiac output are the two most frequently used. Milrinone has a more potent pulmonary and systemic vasodilator effect than dobutamine and is less likely to induce reflex tachycardia (40). As a result, systemic hypotension is a frequent adverse effect of milrinone requiring concomitant vasoconstrictors. Of note, milrinone is cleared almost entirely by the kidneys and therefore renal function must be carefully monitored during use and the milrinone dose adjusted accordingly.

The shorter half-life of dobutamine and its mild vasodilator effect may be consequently preferable in some patients. Exceptions to starting these would include situations where RV failure has been caused by myocardial ischemia and arrhythmia, which may be exacerbated by inotrope use.

Levosimendan infusion also increases RV contractile function and data support the use of this drug in treating RVF of various etiologies (41). Noradrenaline primarily targets the *α*_1_ receptor, causing vasoconstriction with limited *β*_1_ receptor stimulation and cardiac inotropy. However, the *β*_1_ effects on contractility have been shown to improve pulmonary artery/RV coupling in animal models of RV dysfunction (42) hence could be an attractive agent to use in conjunction with an inotropic agent.

**Management of chronic RV failure**

The management of chronic right heart failure follows similar principles to those applied in acute RV failure.

1. Volume management

Diuresis is the mainstay of RHF treatment, using a combination of diuretics (24). The aim of treatment is to reduce congestion and RV volume overload whilst maintaining enough preload to support adequate cardiac output. Regular renal function monitoring is mandatory as excessive volume depletion can lead to pre-renal failure.

2. Afterload management

The causes of increased afterload are heterogenous, and treatment must be directed accordingly. For example, if left sided heart failure with reduced ejection fraction is the cause of pulmonary hypertension and right sided heart failure, optimal guideline-based management including use of beta-blockers, ACE-inhibitors, mineralcorticoid antagonists, ARNis and SGLT-2 inhibitor is indicated (24).

In situations of pulmonary hypertension, the management should treat the specific underlying pathology. Describing the treatment of each subclass of pulmonary hypertension is a subspecialist area and is beyond the scope of this review. Group I PAH are the most complex to manage, and their treatment often constitutes a combination of diuretics, home oxygen (if arterial pO2 < 8 kPa), calcium channel blockers, phosphodiesterase type 5 inhibitors (e.g., sildenafil), prostacyclin analogues (e.g., Eproprostenol) and endotheline receptor antagonists (e.g., Bosentan, Ambisentan).

In Group IV patients suffering from chronic thromboembolic disease, lifelong anticoagulation followed by consideration of pulmonary endarterectomy by a specialist centre may be indicated. Despite their profound therapeutic differences, all these treatment approaches have the same goal, reducing RV afterload.

Similarly, RVF in patients with congenital heart disease, including repaired tetralogy of Fallot, is common but again beyond the scope of this review. Once diagnosed with right heart failure in this context, immediate support should be sought from a specialist adult congenital heart disease team.

2. **Devices**

When optimal medical therapy has not been effective, mechanical circulatory support (MCS) may be beneficial. There are several options that differ in the intensity and duration of support offered, the intended approach of implantation and the need for oxygenation. Current literature is limited to only small prospective randomized trials (43).

(a) **SHORT TERM SUPPORT.** Considerations involved in choosing the most appropriate device depending on the clinical situation are shown in Table 2.
  (i) **Extracorporeal membrane oxygenation (ECMO)**: The first decision to make is deciding between using veno-venous (VV) ECMO and venoarterial (VA) ECMO. VV ECMO only oxygenates and returns blood to the venous system. Therefore, its use is limited to isolated RV failure due to acute hypoxemic respiratory failure. In contrast, venoarterial (VA) ECMO is the preferred option in situations of primary RV injury or RV failure with concomitant LV failure. VA ECMO supports the RV indirectly by reducing preload, reducing RV wall tension, and providing oxygenated blood to the coronary circulation (44). ECMO is especially useful when cardiopulmonary resuscitation is ongoing because it is relatively straightforward to insert peripherally, provides respiratory support and unloads the pulmonary field.
  (ii) **PROTEK DUO:** This cannula-within-a-cannula device allows for percutaneous, single venous access at the right internal jugular vein. The double-lumen cannula consists of a 29 Fr outer cannula and a 16 Fr inner cannula with a length of 46 cm allowing for right atrium drainage. The inlet is positioned in the RA-SVC junction and the return in the pulmonary artery. It can be used with or without an oxygenator in case of respiratory failure (45) and it is connected to an extracorporeal centrifugal pump. It is easy to both position and explant and allows patient mobilisation due to the jugular insertion.
  (iii) **IMPELLA RP:** (Abiomed Inc., Danvers, MA) is a minimally invasive percutaneous microaxial pump used to tackle RV failure. It is used after left ventricular assist device implantation, postcardiotomy or acute RV failure post myocardial infarction (46) with FDA approval in 2017 for up to 14 days. Complications include bleeding at the insertion point and haemolysis or arrhythmias. These are usually due to malpositioning or insufficient support. The Impella RP was first evaluated in the RECOVER RIGHT study showing an improvement in survival and reducing morbidity in patients with RVF following LVAD implantation (43). The post-approval study (PAS) (47) low survival rates (28.6%) in comparison to those in the premarket clinical studies (73.3%) triggered an alert letter released by the Food and Drug Administration (FDA) (48). However, when further analyzing these results, the issue seemed to be frequent off-label Impella RP use, including inappropriate patient selection in the PAS study. The PAS subgroup that would have not qualified for the premarket studies included patients likely to be in cardiogenic shock for more than 48 h, those who had a cardiac arrest, or suffered from a pre-Impella neurological event. This was acknowledged in a more recent FDA update (49).
  (iv) **Extracorporeal magnetically levitated radial pump:** Levitronix® CentriMag® (Levitronix LLC; Waltham, Mass) is a magnetically levitated centrifugal-flow pump designed for temporary extracorporeal support. It can also work with or without an oxygenator. It provides longer support than the devices above mentioned. It is easy to implant and has low risk of thrombosis, reduces pulmonary congestion, and allows for early mobilization. However, its implantation and explant usually require open surgery.
(b) **LONG TERM SUPPORT:** There are limited options for long-term RV support and evidence is lacking. Devices implanted surgically such as HeartWare or HeartMate 3, Berlin Heart or extracorporeal magnetically levitated radial pump (Levitronix) require invasive surgery for both implantation and explantation.
  (i) **RVAD:** In June 3, 2021, Medtronic announced the withdrawal of the HeartWare Ventricular Assist Device (HVAD) from the global market (50). Current data estimates that there are 4,000–5,000 patients on HVAD support in the community, either as bridge for transplantation or destination therapy. The Heartmate 3 (HM3) (Abbott Park, IL) has been recently approved by the FDA for adult heart failure (51). These devices have been used to support chronically impaired RVs but were originally designed for left ventricular failure. The result is that the haemodynamics were designed for a high pressure, low-compliance system and the devices are not necessarily optimized for RV support. Thus, these devices are used off-label and should be reserved for cases where the RV is less likely to recover in the short term, and longer support periods are required. Of note, the timing of RVAD implantation plays a major role in the survival post RVF (52).
  (ii) **Total Artificial Heart (TAH):** it is a biventricular pump that replaces both native cardiac ventricles and all cardiac valves. The SynCardia Total Artificial Heart (TAH, SynCardia Systems, Tucson, AZ) is the only biventricular cardiac replacement approved for bridge to transplantation by the FDA and which carries the European Union CE mark (53).
3. **Heart transplantation**

**Table 2:** Tailored MCS device for right ventricular failure.

	pVA-ECMO	pRVAD	sRVAD	Impella RP
Ongoing CPR	++			
Heamodinamically relevant arrythmias	++			
RV distension + inotropic support	+	+		++
Inotropic support + respiratory acidosis	+	++		
Perioperative RV failure	+	+	++	

pVA-ECMO, peripheral VA-ECMO; pRVAD, peripheral RVAD; sRVAD, systemic RVAD.

Heart transplantation can be considered in cases of advanced refractory chronic heart failure in those situations in which optimized medical treatment, or any other management options have failed.

Exclusion of all reversible causes and assessment of all comorbidities must be established before transplantation can be contemplated.

There are not many situations in which HTx is needed due to pure RV failure. These can be summarized into arrhythmogenic right ventricular cardiomyopathy and extensive ischemia of the right territory.

Although outcomes after isolated heart transplantation are in general exceptional [around 90% survival to 1 year (54)], studies report that the presence of RVAD support before heart transplantation is associated with a relative mortality hazard of 3.03 after transplantation (55). Moreover, preoperative pulmonary hypertension and postoperative RV dysfunction are associated with increased mortality (56).

4. **Special circumstances: LVAD implantation**

RV failure is one of the most encountered complications after durable left ventricular assist device (LVAD). More than 30% of patients experience acute RVF (57), which is the leading cause of premature morbidity and mortality (58).

In the 2-year large MOMENTUM cohort, the prevalence of RV failure was 34% in HeartMate 3 recipients; however, RVAD use was low at 4.1% (59).

The mechanism is based on the sudden increase of preload to the RV after the initiation of the LVAD. The interventricular septum shifts to the left due to the RV dilatation and the effective unloading of the LV caused by a working LVAD causes a detrimental effect on RV contraction (60).

These increases in RV preload after LVAD insertion, though subtle, can also unmask previously subclinical RV dysfunction (61).

Patients on ventilatory support or continuous renal replacement therapy (CRRT) are at high risk for post-LVAD RVF, similarly to patients with slightly increased INR, high NT-proBNP or leukocytosis. High CVP, low RVSWI, an enlarged right ventricle with concomitant low RV strain also identifies patients at higher risk (52).

Post LVAD RFV is a challenging complication with high rates of morbidity and mortality. A minimally invasive approach to LVAD implantation, such as bilateral thoracotomy might play a role in protecting from RV failure (62, 63). This can be due to reductions in blood loss, and preservation of RV geometry due to the fact that the pericardium is not opened completely.

## Future development

The current management of RVF remains suboptimal and leaves much room for improvement. New therapies, both pharmacological and device-centered, should be sought.

It remains of paramount importance to recognize RVF early, understand the underlying etiology and manage appropriately without delays.

Molecular imaging is a developing method based on the existence of specific tracers that bind to molecules of interest. The potential of this method relies in combining targeted molecular imaging with quantification (64). Presently, there is no data showing predictive value for heart failure after MI (65).

Moreover, research is focussing on specific biomarkers [e.g., inflammatory biomarkers (66)] for RV failure, as well as novel hemodynamic indexes.

In a recent study by Wu et al., high-dose dapagliflozin (DAPA) treatment attenuated RV structural remodeling, improved RV function, increased the conduction velocity, restored the expression of key Ca^2+^ handling proteins, increased the threshold for Ca^2+^ and action potential duration alternans, decreased susceptibility to spatially discordant action potential duration (APD) alternans and spontaneous Ca^2+^ events, promoted cellular Ca^2+^ handling, and reduced VA vulnerability in PAH-induced RHF rats. Low-dose DAPA treatment also showed antiarrhythmic effects in hearts with pulmonary hypertension-induced RHF (67).

Despite the great advances in MCS in the last decades, biventricular failure remains a challenge for physicians. Moreover, timing the implantation of the devices remains one of the most important targets in order to achieve good outcomes. Delays in the diagnosis and device implantation is associated with high rates of mortality mostly due to multiorgan dysfunction. Likewise, durable devices specific for the treatment of RVF with applicability to broader patient populations is urgently needed. A recent multicentre study advocates concomitant implantation of MCS for the RV at the time of LVAD implantation, associating it with improved 1-year survival and increased chances of RV support weaning compared to postoperative insertion (68).

## Conclusions

Whereas the right ventricle was once neglected and viewed as a passive player in the circulation, this is not the case anymore, and there has been an increased focus on the diagnosis and treatment of right heart disease over the last two decades. Research has focused on its unique structure and function and has led to the development of both medical and device therapies tackling preload, afterload and myocardial contractility. As with all conditions, the key to the successful management of acute right heart failure is prompt diagnosis to allow timely treatment initiation.

The rapid development of short term MCS over the last decade has certainly facilitated the treatment of RVF. The evolution towards low profile-high output pumps allows the percutaneous treatment of right ventricular failure when medical management does not suffice. These include the minimally invasive percutaneously delivered devices like Impella RP, ECMO and Protek Duo.

Long-term devices and heart transplantation should be reserved for those patients whose RV is unlikely to recover or are likely to require longer support. MCS related mortality is limited to bleeding and device complications, whereas the main morbidity and mortality driver of untreated right heart failure is multiorgan dysfunction (69). Therefore, this gives hope that as devices, implantation techniques and weaning protocols improve over time, mortality will surely follow suit.

## Data Availability

The original contributions presented in the study are included in the article, further inquiries can be directed to the corresponding authors.
